# Compressed-Domain ECG-Based Biometric User Identification Using Compressive Analysis

**DOI:** 10.3390/s20113279

**Published:** 2020-06-09

**Authors:** Ching-Yao Chou, Yo-Woei Pua, Ting-Wei Sun, An-Yeu (Andy) Wu

**Affiliations:** Department of Electrical Engineering, Graduate Institute of Electronics Engineering, National Taiwan University, Taipei 106, Taiwan; endpj@access.ee.ntu.edu.tw (C.-Y.C.); yowoei@access.ee.ntu.edu.tw (Y.-W.P.); willy@access.ee.ntu.edu.tw (T.-W.S.)

**Keywords:** user identification, ECG biometric, compressive sensing, compressive analysis, ECG signal alignment

## Abstract

Nowadays, user identification plays a more and more important role for authorized machine access and remote personal data usage. For reasons of privacy and convenience, biometrics-based user identification, such as iris, fingerprint, and face ID, has become mainstream methods in our daily lives. However, most of the biometric methods can be easily imitated or artificially cracked. New types of biometrics, such as electrocardiography (ECG), are based on physiological signals rather than traditional biological traits. Recently, compressive sensing (CS) technology that combines both sampling and compression has been widely applied to reduce the power of data acquisition and transmission. However, prior CS-based frameworks suffer from high reconstruction overhead and cannot directly align compressed ECG signals. In this paper, in order to solve the above two problems, we propose a compressed alignment-aided compressive analysis (CA-CA) algorithm for ECG-based biometric user identification. With CA-CA, it can avoid reconstruction and extract information directly from CS-based compressed ECG signals to reduce overall complexity and power. Besides, CA-CA can also align the compressed ECG signals in the eigenspace-domain, which can further enhance the precision of identifications and reduce the total training time. The experimental result shows that our proposed algorithm has a 94.16% accuracy based on a public database of 22 people.

## 1. Introduction

**Figure 1:**
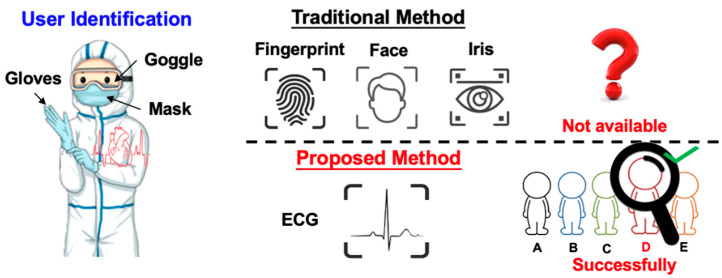
Advantage of the ECG biometric user identification system in certain environments.

Nowadays, user identification plays a more and more important role for authorized machine access and remote personal data usage. For reasons of privacy and convenience, biometrics-based user identification has already become the mainstream method since they are more secure than traditional identification methods, such as passwords and ID cards. Therefore, we need an effective, reliable, and convenient biometric system [1,2]. However, most of the biometric methods can easily be imitated or artificially cracked. For instance, voices could be recorded, fingerprints could be recreated in latex, and personal photos can trick facial recognition. Therefore, in recent days, new forms of biometrics, such as electrocardiography (ECG), are used as biometrics signatures [3]. In addition, the ECG signal also indicates the vital status of the user. The advantages of ECG biometric user identification are as follows. Firstly, the ECG signal is continuous and difficult to be imitated, and the system cannot be cheated using artificial signals. Secondly, some scenarios are even more suitable for ECG-based user identification (say, via Bluetooth transmission). For instance, in the cleanroom or IC fabs, or in pandemic-control negative-pressure isolation rooms, most staff are wearing isolation gowns, so it is impossible to take off the mask and gloves to pass the security check using conventional methods. In the aforementioned cases, it is more proper to apply wireless-transmitted ECG signals to verify if the person has granted permission, as shown in Figure 1.

**Figure 2:**
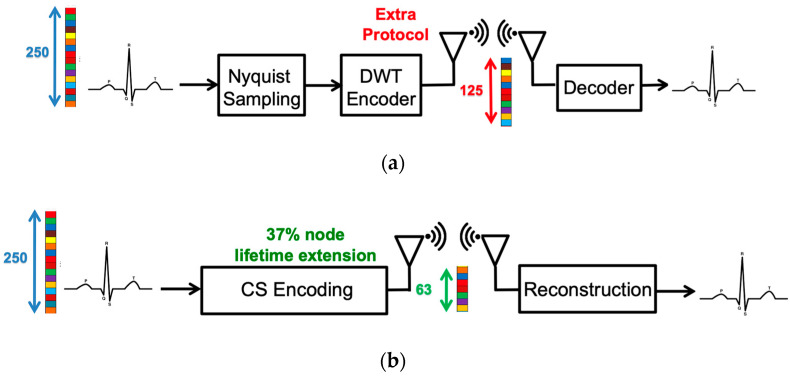
The procedure of (**a**) conventional measured-and-compressed techniques and (**b**) compressive sensing technique.

Although ECG biometric user identification can provide higher security, ECG sensors used for long-term detection demands high power consumption. Fortunately, the emergence of compressive sensing (CS) technology alleviates the power problem of the ECG sensor. CS is a technique that combines both sampling and compression via random projections to reduce the power of data acquisition and transmission [4,5,6]. The conventional compressed data transmission is shown in Figure 2a. The ECG signals are sampled at the Nyquist rate. The Daubechies Wavelet Transform (DWT) is applied to the ECG signal at the transmitter side. Then, at the receiver side, the inverse DWT (IDWT) is used to reconstruct the ECG signals. The whole sensing processing requires lots of computing resources and memory. On the other hand, the CS algorithm only measures the ECG signals with fewer measurements and reconstructs signals from these fewer measurements, as shown in Figure 2b. Furthermore, in CS-based compression of the ECG signal, additional compression hardware is not required. Compared with the DWT technology, the CS-based ECG sensor can extend the battery lifetime by 37.1% [7]. Application of the CS technique to wearable e-health devices provides huge savings and an improvement in the power consumption domain. In [8], it is shown that, in the applications of long-term telemonitoring systems, wireless biomedical sensor nodes are known to be resource-limited. Hence, it is a crucial problem to reduce the signal acquisition on these sensing systems and enhance the energy efficiency of data transmission. Therefore, applying CS sampling results in the lifetime extension of the sensor node and makes the technique especially attractive in long-term telemonitoring systems. In addition to transmission power reduction, several researches had shown that CS can also be a built-in encryption technique [9,10]. It has been shown that the random sampling feature of the CS algorithm can bare embedded privacy and encryption features with negligible overheads. Therefore, CS-based ECG biometric user identification is more suitable for practical considerations, such as wearable devices.

Nevertheless, the CS-based biometric system is still facing the challenge of altering the signal features and the high complexity of data analytics. The current state-of-the-art CS-based ECG signal classification systems are reconstruction learning (RL) and compression learning (CL) [11,12]. In the reconstructed learning (RL) algorithm, it reconstructs the original ECG signal from the compressed ECG signal before performing identification using the CS reconstruction algorithms, such as Basis Pursuit (BP) [13] and Orthogonal Pursuit (OMP) [14]. Nevertheless, the reconstruction process consumes a lot of computing resources, resulting in some problems for signal analysis in reconstructed learning. Previous research pointed out that the processing time for reconstruction spans almost the entire processing time. Furthermore, the reconstruction of the compressed ECG signal also destroys the privacy obtained from the CS algorithm. It will put the original ECG signal at risk of being exposed. 

**Figure 3:**
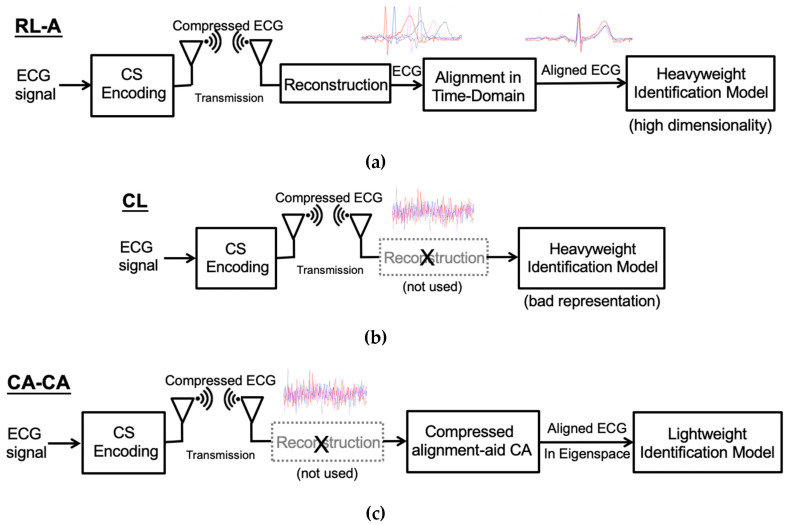
(**a**) The reconstructed learning with alignment (RL-A) algorithm. (**b**) Compressed learning (CL) algorithm. (**c**) Our proposed compressed alignment-aided compressive analysis (CA-CA) algorithm.

Normally, the ECG wave of a normal heartbeat consists of a P wave, a QRS complex, and a T wave. Each wave provides different information, such as the P wave representing atrial depolarization, the QRS complex representing ventricular depolarization, and the T wave representing ventricular repolarization. However, if these waves are not aligned, the information carried by these waves will become blurred. Note that the original ECG signal retains the time-domain characteristics, so it can be easily aligned. As shown in Figure 3a, before alignment, the differences between heartbeats are significant. Therefore, the ECG signal should be aligned to reduce the difference for a more accurate analysis [15], as shown in Figure 3a. Since the ECG signals are aligned, the model can quickly learn more detailed information about the signals, which can perform better ECG biometric user identification. The reconstructed learning with alignment (RL-A) algorithm is shown in Figure 3a. 

On the other hand, the compressed learning algorithm bypasses the reconstruction process, analyzing directly in the compressed domain, as shown in Figure 3b. After CS compression, although the information of identification is preserved, the representation will degrade learnability, leading to higher model complexity in the compressed domain [16]. Therefore, a large number of learning resources are needed to learn the compressed ECG signals directly. Besides, it is difficult for compressed learning to process signals in the compression domain. The time-domain ECG signal alignment only requires shifting the ECG signal. Hence, we can efficiently handle the raw ECG signal, such as feature extraction and alignment. Nevertheless, the compressed signal cannot simply shift the ECG signal to achieve alignment. After the CS compression, the ECG signals lose its time-domain characteristics; the five waves of the ECG signal are all destroyed, as shown in Figure 3b. Therefore, we propose a compressed alignment-aided compressive analysis (CA-CA) algorithm to remove the reconstruction and align the compressed ECG signal in the eigenspace domain directly. Figure 3c shows the overall block diagram of our algorithm. In Table 1, it is shown that the complexity and performance of the different frameworks are qualitatively compared. Our proposed CA-CA algorithm can take care of both complexity (time) and performance (accuracy), which will be further verified in Section 4 and Section 5. Our main contributions are summarized as follows:

- *We propose a compressive analysis (CA) with a PCA-assisted dictionary to directly and more effectively learn from the compressed signals without reconstruction*: Conventional CS-based ECG biometric user identification requires reconstructing the compressed ECG signal into the original raw signal. The back-end signal reconstruction makes the entire identification system highly complex. We propose a compressive analysis algorithm by using the principal component analysis (PCA)-based dictionary in the compressed domain.
- *We propose a low complexity compressed alignment-aided compressive analysis (CA-CA) algorithm to remove the reconstruction and align the compressed ECG signal in the eigenspace-domain*: Aligning ECG signals can reduce the differences between the ECG signals, thereby improving identification accuracy and reducing learning resources. However, conventional ECG signal alignment methods are only valid in the time domain. Therefore, we propose a compressed alignment-aided compressive analysis (CA-CA) algorithm to remove the reconstruction and align the compressed ECG signal in the eigenspace domain directly. Our proposed algorithm can have almost equal precision of identifications, but reduce the overall complexity compared with a conventional RL-A algorithm.

The remainder of this paper is organized as follows. Section 2 reviews the related algorithms and prior works in ECG signal classification. Section 3 details our proposed algorithm. Section 4 demonstrates the simulation results of our proposed algorithm. In Section 5, we analyze the computation time and memory overhead. Finally, we conclude our work in Section 6. 

## 2. Related Works

### 2.1. Compressive Sensing (CS)

Compressive sensing [4,5,6] is a revolutionary technique, which allows acquiring and compressing signals simultaneously. If the signal is sparse in a specific domain, CS can guarantee an almost perfect reconstruction even with fewer measurements than the Nyquist rate requirement. The random sampling process can be formulated as a multiplication of the sensing matrix Φ and the original signal x. As a result, we acquire the measurement $\hat{x}$ as

(1) $$\hat{x}=\Phi x$$

where $\hat{x}\in {\mathbb{R}}^{M},\Phi \in {\mathbb{R}}^{M\times N},x\in {\mathbb{R}}^{N}$. The entries of the CS measurement matrix can be chosen randomly as long as the column in Φ is incoherent with the basis in which the signal has a sparse representation. Since the dimension of the measurement $\hat{x}$ is smaller than the dimension of the original signal **x**, this process is regarded as compressive sensing.

The CS reconstruction algorithms are performed to recover the signal from the measurement $\hat{x}$ and sensing matrix Φ. Since natural signals are often not sparse in the time domain but sparse with the proper projection basis Ψ, CS divides the signal estimation into two procedures. First, we find the sparse solution by solving

(2) $$\underset{s}{min}{∥s∥}_{0}=\overset{N}{\underset{i=1}{\sum }}{\left|s_{i}\right|}_{0}\;subject\;to\;\hat{x}=\Phi x=\Phi \Psi s=\Theta s$$

where **s** is the sparse coefficient vector of signal x projected on Ψ and ${∥s∥}_{0}$ is the $l_{0}$ norm of s. Secondly, the time domain’s reconstructed signal x is estimated by

(3) x=Ψs

**Figure 4:**
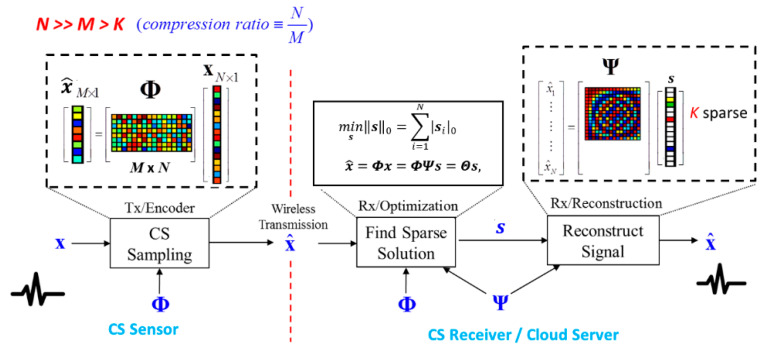
Overall framework of the compressive sensing (CS) technique.

Since CS reconstruction is an underdetermined question, algorithms based on linear programming, such as basis pursuit (BP) [13], can find an optimal solution of Equation (2). However, the computational complexity is exceptionally high. There are some greedy-type algorithms, such as orthogonal matching pursuit (OMP) [14], that have been proposed to accelerate the process of reconstruction. The overall framework of CS is shown in Figure 4.

### 2.2. Reconstructed Learning (RL) 

The prior art of signal analysis for the CS-based ECG signal classification algorithm is reconstructed learning (RL) [17,18]. There are two phases: training and analysis. We can first utilize part of the ECG signal as the training data to train the machine learning models for biometric user identification [3] and the dictionary for sparse representation. After training, the ECG signal is measured by the wearable CS sensor. After the receiver reconstructs the compressed ECG signal back to the original waveform, it can be further classified by the pre-trained machine learning model. 

There are two main blocks in the reconstructed learning algorithm. The CS block defines the sparse dictionary and accomplishes the reconstruction function. For perfectly reconstructing the original ECG waveform, the purpose of the sparse dictionary is to guarantee the sparsity of signals. One possible solution of the dictionary is the predefined dictionary, like the discrete cosine transform (DCT) or DWT. Nevertheless, if the sparsity on the predefined basis is not enough for reconstruction, there exist some algorithms for dictionary learning, such as K-SVD [19] and the method of optimal directions (MOD) [20]. These algorithms learn the sparse dictionary from a specific training dataset. The popular reconstruction algorithms are BP, OMP, and stochastic gradient pursuit (SGP) [21].

The purpose of the machine learning block is user identification. Famous machine learning algorithms, including SVM [22] and neural networks [23,24], have been widely adopted for ECG signal classification or disease detection. They identify the similar characteristics between data within the same class and the significant differences between data within the different classes.

### 2.3. Compressed Learning (CL)

The compressed learning (CL) algorithm directly performs the inference, such as classification or regression, in the compressed domain, which is beneficial from both a compressive sensing and machine learning point of view. From the CS viewpoint, it eliminates the abundant cost of recovering irrelevant data; in other words, compressed learning is like a sieve and makes it possible to only recover the desired signals. From the ML viewpoint, CS can be regarded as an efficient universally sparse dimensionality reduction from the data domain to the measurement domain.

**Figure 5:**
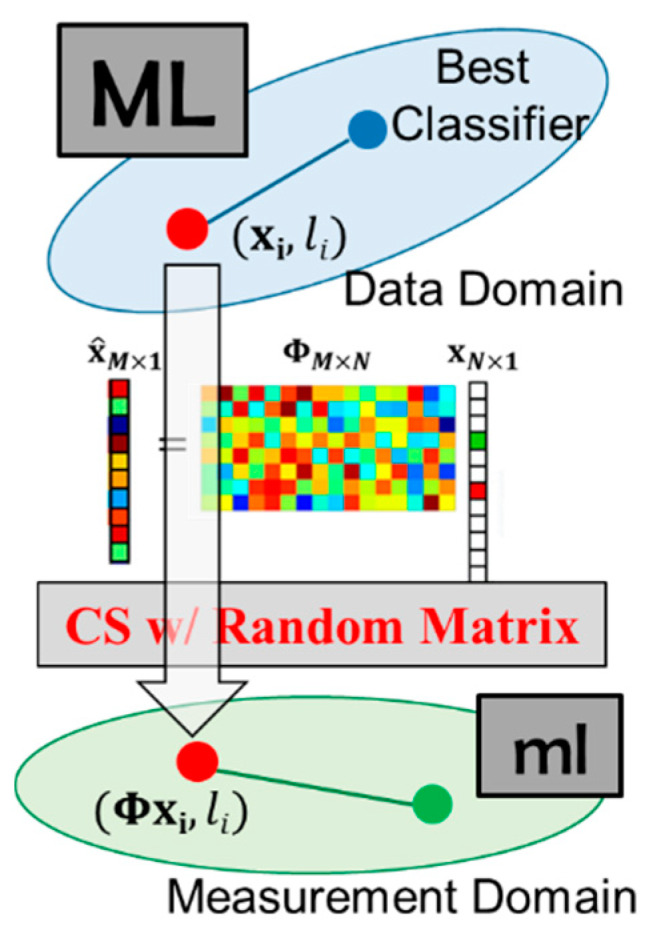
CS can persevere in the data structure.

Random projections (RP) used in CS have been used in real-time applications to reduce the computation latency [25]. RP provides feasible solutions to the well-known Johnson–Lindenstrauss lemma (JLL) [26]. Thus, the distances between the points are approximately preserved as mapping down onto a lower dimensional space. There are also some theoretical works focusing on deriving the error bounds for classifiers with random and compressed features. In [27], they proposed the idea of compressed learning, which is precisely the same as compressive analysis without reconstruction. They used JLL to derive an error bound for learning discriminative models directly in the measurement domain, using a support vector machine (SVM) classifier with linear kernels. They proved that if the instances are provided directly in the measurement domain, the soft margin SVM’s classifier trained with compressively sampled training data has almost the same performance as the best possible classifier in high dimensional space, as shown in Figure 5. In addition to removing the abundant cost of reconstruction, the computational cost of machine learning can be reduced in the low dimensional measurement domain. In compressed learning, it bypasses the reconstruction process and analyzes directly in the measurement domain. Since the distances between the points are retained by Johnson–Lindenstrauss lemma (JLL), the learnability is preserved [26]. However, due to random sampling, the time-domain characteristics of the ECG signal will be disrupted. As a result, CL cannot directly align the ECG signal by the compressed ECG signal. This leads to the ML model being unable to learn more detailed information about the signal.

## 3. Compressed-Domain ECG Biometric User Identification Using Compressive Analysis

**Figure 6:**
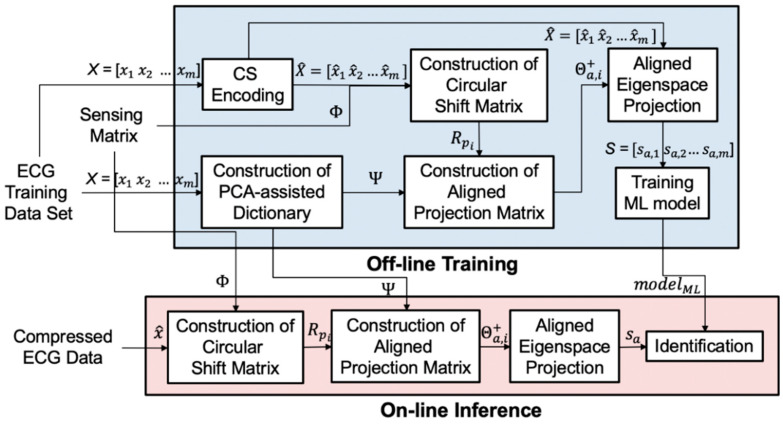
The overall block diagram of our proposed algorithm with a PCA-assisted dictionary.

In this section, we detail our algorithm. As shown in Figure 6, it contains two stages: (1) the offline training stage and (2) the online inference stage. The first stage is the offline training phase operated in the time domain. We train the dictionaries Ψ using the PCA algorithm. The goal of these dictionaries is to preserve the necessary information of the training dataset with minimum components. The second stage is the online inference phase in the compressed domain. The aligned eigenspace projection matrix is built by the PCA dictionary Ψ, sensing matrix Φ, and circular shift matrix $R_{p}$. The compressed signals are transformed by the aligned eigenspace projection matrix. The aligned projected data $s_{a}$ is analyzed by a pre-trained classifier. 

### 3.1. CS-Based PCA Dictionary Learning

The goal of principal component analysis (PCA) [28] is to determine the most meaningful basis for re-expressing the data sets. PCA utilizes the property that the intrinsic dimension of a data set is much smaller than the data dimension. The data set can thus be represented by a small number of bases, also known as principal components (PC). Principal components are linear transformations of the original set of variables that are orthogonal and ordered by the variety of data on the corresponding basis. By choosing the first few principal components as the basis, we can transform the data set onto a lower-dimensional subspace while retaining the most information. In addition, because of the nature of the ECG signal, only a few waves contain the necessary information, as shown in Section 1, and most signal values are close to zero. Therefore, a PCA algorithm is suitable for ECG signal analysis; it will reduce the ECG signal dimension without undermining user identity information.

**Figure 7:**
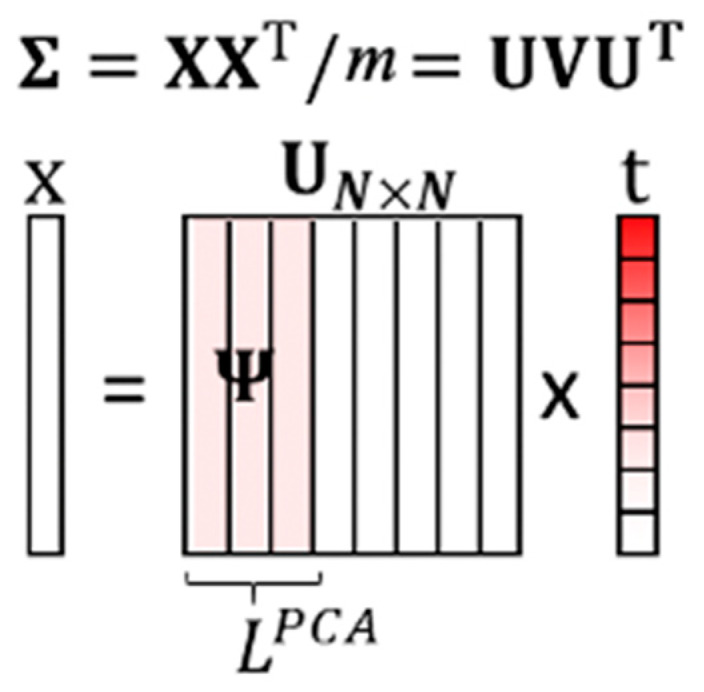
The illustration of the training PCA-based dictionary.

The first step of a PCA is to calculate the covariance matrix Σ of the data set $X=\left[x_{1}\;x_{2}\ldots \;x_{m}\right]$, where $x_{i}\in {\mathbb{R}}^{N}$ and m is the number of data in the data set:

(4) $$\Sigma =\frac{1}{m}\left(X-\bar{x}h\right){\left(X-\bar{x}h\right)}^{T}$$

where $\bar{x}$ is the mean of each row of X, h is a 1xm vector of all 1s, and ${\left\{\cdot \right\}}^{T}$ denotes the transpose. The final step of the PCA is to perform eigenvector decomposition on Σ:

(5) $$\Sigma =UVU^{T}$$

where U is the eigenvectors of Σ, and V is the eigenvalues of Σ. Through the PCA, the eigenvectors are sorted by the magnitude of eigenvalues, which represent the variance on the eigenvectors. So, we can get the eigenvector ordering, which can represent the data set information. It is shown in [28] that with a high signal-to-noise ratio (SNR), large variances have an essential structure, while low variances represent noise. Therefore, by choosing the first L important principal components, the primary data structure can be kept, and the noise can be mitigated in the low dimensional subspace, as shown in Figure 7. 

A data vector x can be re-expressed as Ut, where t is the transformed vector. The PCA-based dictionary Ψ is built by the first $L^{PCA}$ columns of U:

(6) $$\Psi =U\left(:,1:L^{PCA}\right)$$

The PCA-assisted dictionary algorithm is summarized in Algorithm 1. The rest eigenvectors are considered as noise. The PCA-based dictionary keeps the most critical information with the least bases. We utilize the data statistics information to eliminate the redundant basis and build an overdetermined dictionary.
**Algorithm 1**. PCA-D: Ψ= PCA-D ($X,L^{\mathrm{PCA}}$)**Input:** N-dimensional training set $X=\left[x_{1}\;x_{2}\;\ldots \;x_{m}\right]$ and the PCA-assisted dictionary length $L^{\mathrm{PCA}}$ (with $L^{\mathrm{PCA}}$ ≤ *N*), where $X\in {\mathbb{R}}^{N\times m}$Compute the mean $\bar{x}=\frac{1}{m}\overset{m}{\underset{i=1}{\sum }}x_{i}$, where $\bar{x}\in {\mathbb{R}}^{N\times 1}$Compute the covariance matrix $\Sigma =\frac{1}{m}\left(X-\bar{x}h\right){\left(X-\bar{x}h\right)}^{T}$, where h is a  1×m vector of all 1sFind the eigenvector decomposition on $\Sigma =UVU^{T}$, obtaining the eigenvectors U$\Psi =U\left(:,1:L^{\mathrm{PCA}}\right)$**Output:** PCA-assisted dictionary Ψ

For the data set in which the intrinsic dimension of the data set is much smaller than the data dimension, the variance of most principal components is close to zero. This means the data set is sparse in the eigenspace. Therefore, we can find a low dimensional model with a fixed basis in the sparse space and preserve the most information at the same time. The PCA-assisted dictionary is proposed by utilizing the sparsity in the eigenspace. The general dictionary learned by MOD [20] is replaced by the basis formed by the first $L^{PCA}$ principal components. $\hat{x}\;$ can be re-expressed as

(7) $$\hat{x}=\Phi \Psi s=\Theta s$$

We can further solve the transformed metadata s in the least square sense and obtain:

(8) $$s={\left(\Theta^{T}\Theta \right)}^{-1}\Theta^{T}\hat{x}$$

The PCA-assisted dictionary is used for sifting the sub-eigen information from the CS measurements online, and it is built by eigenspace learning offline. The algorithm can reduce the memory overhead with a single lightweight machine learning model and the computational complexity with sifting by a matrix-vector product rather than sparse coding. Instead of finding support atoms by sparse coding, we utilize the statistics information and only focus on the bases formed by the important eigenvalues. Conventional dictionary learning methods exploit the benefits of sparsity and seek to find the sparest representation of data [20]. The transformation to metadata evolves sparse coding to find the corresponding atoms iteratively. The computation overhead is $O\left(M^{2}N^{1.5}\right)$ according to [17]. This is impractical for online usage. Therefore, we utilize the data statistics information to eliminate the redundant basis and remove the iterative operation. With the assumption of CS theory, the compressed signal can be projected to the low dimensional subspace with a small error if the data has sparsity on certain bases. The information of data with sparsity in the eigenspace is preserved by transformation via a PCA-assisted dictionary. The transformation overhead is then reduced to a matrix multiplication, which is $O\left(L^{\mathrm{PCA}}M\right)$.

### 3.2. Offline Training

The training phase is operated offline. It consists of two stages: (1) construction of the PCA-assisted dictionary Ψ and (2) identification of model training. The offline compressive analysis algorithm is summarized in Algorithm 2.
**Algorithm 2**. CA Offline: $\left[model_{ML},\;\Psi \right]=$ CA-Off $\left(X,L^{\mathrm{PCA}},\Phi \right)$
**Input:** N-dimensional training set $X=\left[x_{1}\;x_{2}\;\ldots \;x_{m}\right]$, the PCA-assisted dictionary length $L^{\mathrm{PCA}}$ (with $L^{\mathrm{PCA}}\;\leq$ *N*), and sensing matrix ΦCompute the PCA-assisted dictionary Ψ= PCA-D ($X,L^{\mathrm{PCA}}$)Compressed the input signal $\hat{X}=\Phi X$ (transmitter)Compute the eigenspace projection matrix $\Theta^{+}={\left(\Theta^{T}\Theta \right)}^{-1}\Theta^{T}$, where Θ=ΦΨCompute the training metadata set $s=\Theta^{+}\hat{x}$, $s\in {\mathbb{R}}^{L^{PCA}\times m}$Training the ML classification model $model_{ML}$ from training metadata set s**Output:** ML classification model $model_{ML}$ and PCA-assisted dictionary Ψ

We use the ECG training dataset to generate the PCA dictionary. The dataset can be collected from different people with the same measuring instrument. The ECG signal has a standard PQRST wave. After PCA, the first few dimensions of the principal components with larger energy are retained, which means that the information of the PQRST wave is retained. Since the dictionary can be trained by the ECG signals from different people, all the individuals can share the same PCA dictionary. We can store the dictionary directly into the wearable device, which significantly improves usability. In the identification model training stages, the compressed ECG signal is used to obtain sub-eigen information through eigenspace projection. Finally, the system passes the information to the machine learning model, which means the identification model will be trained.

### 3.3. Online Inference

**Figure 8:**
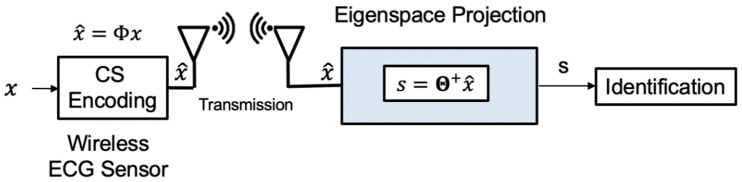
The online identification of our proposed algorithm.

This phase is implemented online, as shown in Figure 8. CS compresses the input signal. We avoid reconstruction to reduce most computation overhead. But the signal still retains some parts that are not related to inference. Therefore, we develop a compressed signal processing algorithm that uses the PCA dictionary formed during the training phase to project the compressed input data onto a low-dimensional and most informative signal subspace. After the compressed ECG signal is reduced by the projection dimension of the PCA dictionary and then using of the offline-trained SVM model, the user can be determined. The algorithm can reduce the memory overhead with a single lightweight machine learning model and the computational complexity with sifting by a matrix-vector product rather than sparse coding. The eigenspace projection matrix is built by the PCA dictionary Ψ and sensing matrix Φ. The eigenspace projection matrix transforms the compressed signals. The projected metadata s is further analyzed by a pre-trained classifier. The online compressive analysis algorithm is summarized in Algorithm 3.
**Algorithm 3**. CA Online: y= CA-On $\left(\hat{x},\Phi ,\;\Psi ,\;model_{ML}\right)$
**Input:** M-dimensional compressed signal $\hat{x}$, sensing matrix Φ, PCA-assisted dictionary Ψ, and ML classification model $model_{ML}$Compute the metadata $s=\Theta^{+}\hat{x}$, where Θ=ΦΨPrediction $y=ML\;\left(s,model_{ML}\right)$**Output:** Prediction of y

### 3.4. Compressed Alignment of the ECG Signal

A typical ECG wave of a normal heartbeat consists of a P wave, a QRS complex, and a T wave. However, if these waves are not aligned, the information carried by these waves will become blurred. The ECG signal should be aligned to reduce the difference for a more accurate analysis [15]. Aligning the ECG signal in the time-domain is not difficult, just find the alignment point and rotate it. The equation can be expressed as

(9) $$x_{a}=Rx$$

where x is the unaligned/raw ECG signal, $x_{a}$ is the aligned ECG signal, and R is the circular shift matrix. The alignment point used here is the maximum amplitude in the ECG signal, called R-peak. 

**Figure 9:**
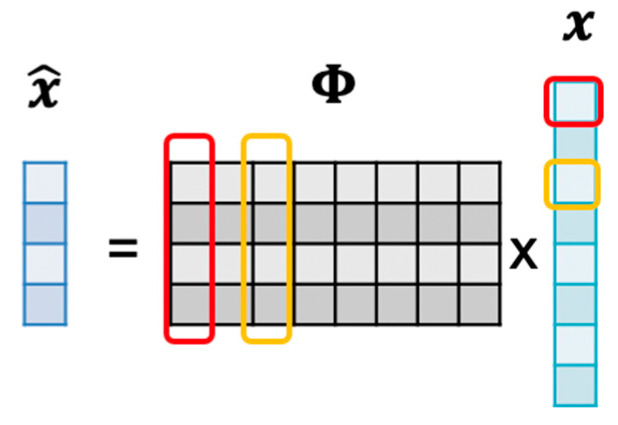
The correlation between Φ and x comes from $\hat{x}$.

The compressed signal cannot be simply multiplied by a circular shift matrix to achieve the alignment. The main reason is that through CS random sampling, the five waves of the ECG signal is also disrupted. Therefore, we propose a new algorithm that can be used for compressed-domain alignment to reduce the power consumption and improve the accuracy. First, we need to select the reference point for alignment. Although it is simple to find the ECG peak directly from the time-domain signal, it is not easy to find the R-peak from the CS compression signal. We look for approximate reference points in a low-complexity way. In the first step, we simply extend Equation (1):

(10) $$\hat{x}=\Phi x=\left[c_{1}\;c_{2}\ldots c_{N}\right]{\left[x_{1}\;x_{2}\ldots \;x_{N}\right]}^{T}\;\;=x_{1}c_{1}+x_{2}c_{2}+\ldots +x_{N}c_{N}$$

where $c_{i}$ is the ith column of Φ, and $x_{j}$ is the jth entry of x. It can be known from the Equation (10) that the compressed signal $\hat{x}$ is obtained by each point of the ECG signal and each column of Φ, as shown in Figure 9.

**Figure 10:**
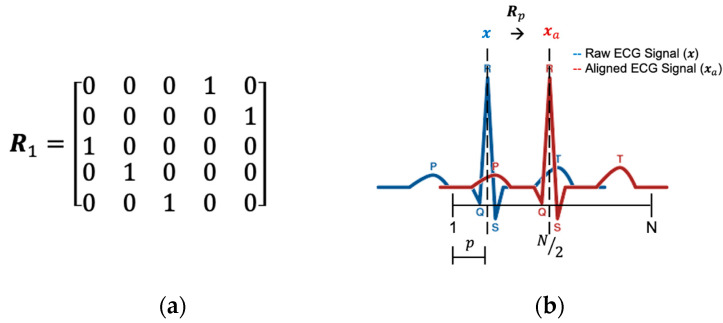
(**a**) Circular shift matrix $R_{p}$ when N = 5 and *p* = 1. (**b**) The relationship between $R_{p}$ and *p*.

Therefore, we can estimate the coordinate of the R-peak by the correlation of Φ and $\hat{x}$:

(11) $$p≅max_{index}\left(\Phi^{T}\cdot \hat{x}\right)$$

where $\Phi^{T}$ is the transpose of Φ. As mentioned above, R-peak is used as the reference point because low-complexity correlation methods can quickly estimate it. We make a tradeoff between the complexity and the exact coordinate of the ECG peak. Finally, we use a low-complexity method to find the approximation p. After obtaining p from Equation (11), we can generate the circular shift matrix $R_{p}$ based on the corresponding reference point coordinate p:

(12) $$R_{p}=\{\begin{array}{c} CirS\left(I_{N\times N},\lceil N/2\rceil -p\right)\;\;\;\;\;\;\;\;\;,\;\;p\leq \lceil N/2\rceil \\ CirS\left(I_{N\times N},\lceil N/2\rceil -p+N\right),\;\;p>\lceil N/2\rceil \end{array}\;\;$$

where $I_{N\times N}$ is the N×N identity matrix, ⌈·⌉ is the ceiling function, and CirS(A,B) is the row circular shift function (up-to-down) which shifts the A matrix B times. For example, if **N** = 5 and **p** = 1, then the circular shift matrix $R_{p}$ as shown in Figure 10a. Since $R_{p}$ is shifted by the identity matrix, it is also an orthogonal matrix. The abridged general view of the $R_{p}$ and p is shown in Figure 10b. 

By using Equation (6), we replace the traditional dictionary learning method with a PCA. The PCA-based dictionary Ψ is constructed from the first L columns of eigenvector U. PCA-based dictionary keeps the most important information with the least vector. We first assume the aligned ECG signal $x_{a}$ has below representation $x_{a}=\Psi s_{a}$. Therefore, we can re-represent Equation (1) as

(13) $$\hat{x}=\Phi x=\Phi \cdot R_{p}^{T}x_{a}=\Phi \cdot R_{p}^{T}\cdot \Psi s_{a}=\Theta_{a}s_{a}$$

where $x=R_{p}^{T}x_{a}$, $\Theta_{a}=\Phi \cdot R_{p}^{T}\cdot \Psi$, and $s_{a}$ is the representation vector of $x_{a}$. The representation vector $s_{a}$ is obtained by

(14) $$s_{a}=\Theta_{a}^{+}\hat{x}$$

where ${\left\{\cdot \right\}}^{+}$ denotes the pseudo-inverse. Finally, we further implement the training and inference of machine learning models in the $s_{a}$.

### 3.5. Proposed CA-CA Algorithm

**Figure 11:**
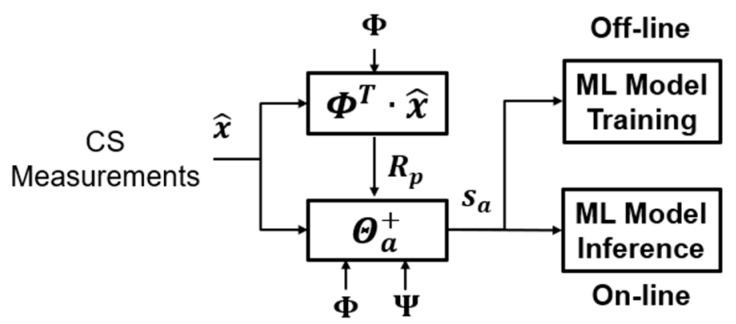
The block diagram of the compressed alignment-aided compressive analysis algorithm.

The detailed step and virtual code are shown in Algorithm (4). The block diagram is in Figure 11.

1. Find the coordinate of the reference point p from the measurement signal.
2. Generate a circular shift matrix $R_{p}$ based on the corresponding reference point coordinate p.
3. Express the compressed ECG signals on the same basis based on the PCA and $R_{p}$.
4. Train the ML models and classification using the representation vectors generated from Step 3.

The offline and online compressed-domain alignment-aided compressive analysis algorithm is summarized in Algorithms 4 and 5, respectively.
**Algorithm 4**. CA-CA Offline: $\left[model_{ML-A},\;\Psi \right]=$ CA-CA-Off $\left(X,L^{\mathrm{PCA}},\;\Phi \right)$
**Input**: N-dimensional training set $X=\left[x_{1}\;x_{2}\;\ldots \;x_{m}\right]$, the PCA-assisted dictionary length $L^{\mathrm{PCA}}$ (with $L^{\mathrm{PCA}}$ ≤ *N*), and sensing matrix ΦCompute the PCA-assisted dictionary Ψ= PCA-D ($X,L^{\mathrm{PCA}}$)Compressed the input signal $\hat{X}=\Phi X$ (transmitter)for i from 1 to m do4.1.Find the reference point index $p_{i}≅max_{index}\left(\Phi^{T}\cdot {\hat{x}}_{i}\right)$4.2.Compute the circular shift matrix $R_{p_{i}}$ with $p_{i}$4.3.Compute the aligned eigenspace projection matrix $\Theta_{a,i}^{+}={\left(\Theta_{a,i}^{T}\Theta_{a,i}\right)}^{-1}\Theta_{a,i}^{T}$, where $\Theta_{a,i}=\Phi \cdot R_{p_{i}}^{T}\cdot \Psi$4.4.Compute the aligned metadata $s_{a,i}=\Theta_{a,i}^{+}{\hat{x}}_{i}$4.5.Collect the training metadata set $S_{a}\left(:,i\right)=s_{a,i}$Training the ML classification model $model_{ML-A}$ from training metadata set $S_{a}$**Output**: ML classification model $model_{ML-A}$ and PCA-assisted dictionary Ψ

**Algorithm 5**. CA-CA Online: y= CA-CA-On $\left(\hat{x},\;\Phi ,\;\Psi ,model_{ML-A}\right)$

**Input**: M-dimensional compressed signal $\hat{x}$, sensing matrix Φ, PCA-assisted dictionary Ψ, and ML classification model $model_{ML-A}$Find the reference point index $p≅max_{index}\left(\Phi^{T}\cdot \hat{x}\right)$Compute the circular shift matrix $R_{p}$ with pCompute the aligned eigenspace projection matrix $\Theta_{a}^{+}={\left(\Theta_{a}^{T}\Theta_{a}\right)}^{-1}\Theta_{a}^{T}$, where $\Theta_{a}=\Phi \cdot R_{p}^{T}\cdot \Psi$Compute the aligned metadata $s_{a}=\Theta_{a}^{+}\hat{x}$Prediction $y=ML\;\left(s_{a},model_{ML-A}\right)$**Output**: prediction y


## 4. Simulation Result

### 4.1. Simulation Setting

The simulation settings are summarized in Table 2, including the processor configurations and the data parameters. There are two popular open databases for evaluating ECG user identification performance, namely the QT and ECG-ID database. There is a serious data imbalance problem in the ECG-ID dataset; that is, the smallest data of the ECG-ID database only has 20 s, but the largest data of the ECG-ID database is 440 s. 

Generally speaking, the mean value of the ECG-ID database is 68 s, and the standard deviation is 63 s. Therefore, in using our proposed linear algorithm, the balance between the evaluated data will have a serious impact on the performance. This is the main reason why we did not choose the ECG-ID database to evaluate the performance. The QT database contains both normal and cardiological disorder ECG signals, with a balanced distribution. Therefore, all annotations were manually reviewed in order to select records without cardiological disorders. From [30], records of normal ECG rhythms were selected, and we continued to use them. The following records were selected: sel103, sel117, sel123, sel16265, sel16272, sel16273, sel16420, sel16483, sel16539, sel16773, sel16786, sel17152, sel17453, sel301, sel302, sel306, sel307, sel310, sele0111, sele0124, sele0133, and sele0210. The Physionet QT database is available in [31]. The database we selected contains 22 ECG recordings obtained from 22 persons. Each record was divided into 850 data sets, including 600 training data sets and 250 testing data sets. The amplitude of each ECG signal was normalized to −1–1 mV/mV to reduce the difference between the measuring instruments. Model selection for the SVM was performed by a cross-validated grid search.

### 4.2. Performance under the Compressed Domain Without Alignment

We compared our algorithm with reconstructed learning and compressed learning. For reconstructed learning, we first reconstructed signal x from $\hat{x}$ before training the machine learning model. For compressed learning, we trained the learning model directly on $\hat{x}$. In the proposed CA framework, we trained the PCA-based dictionary from the ECG signals and computed the eigenspace projection matrix. Finally, we trained the learning model on metadata s.

In reconstructed learning, a dictionary (sparsify matrix) is required to reconstruct the signal. In compressed learning, no dictionary is required, as learning is directly on the compressed data. In the proposed compressive analysis, a dictionary (projection matrix) is required to project the signal to the eigenspace. As shown in Table 3, the results show that the accuracy of the ECG identification system is over 85% in all the CS-based algorithms. Compared with reconstructed learning, our compressive analysis algorithm can not only maintain the same accuracy range, about 87%, but also reduce the dictionary size from 250 × 250 to 250 × 39. The compressed learning does not use a dictionary for projection and reconstruction, but its accuracy is similar to that of reconstructed learning and compressive analysis. Therefore, from the simulation result, we know that the CS-based compressed-domain ECG biometric user identification system is feasible. 

### 4.3. Performance under the Compressed Domain with Alignment

Here, we used the same simulation settings as in Table 2. We compared our algorithm with reconstructed learning with alignment and compressed learning. The reconstructed learning with alignment algorithm is slightly different from the reconstructed learning. Compared with reconstructed learning, reconstructed learning with alignment align the ECG signals before identification. In addition to analyzing the learning performance and resource overheads, we also analyzed the computation time. The results are shown in Table 4. 

**Figure 12:**
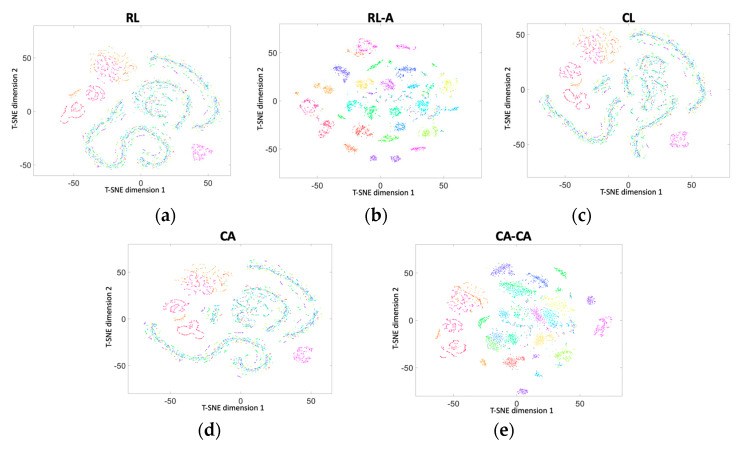
Two-dimensional t-SNE (t-distributed random neighbor embedding) visualization of the data in (**a**) RL, (**b**) RL-A, (**c**) CL, (**d**) our proposed CA, and (**e**) our proposed CA-CA.

Next, we used two-dimensional t-SNE (t-distributed random neighbor embedding) [32] to visualize the accuracy, as shown in Figure 12. Colors denote users, and each point represents the data projected from the different dimensions into two dimensions. It presents the identification capabilities of different frameworks. 

**Figure 13:**
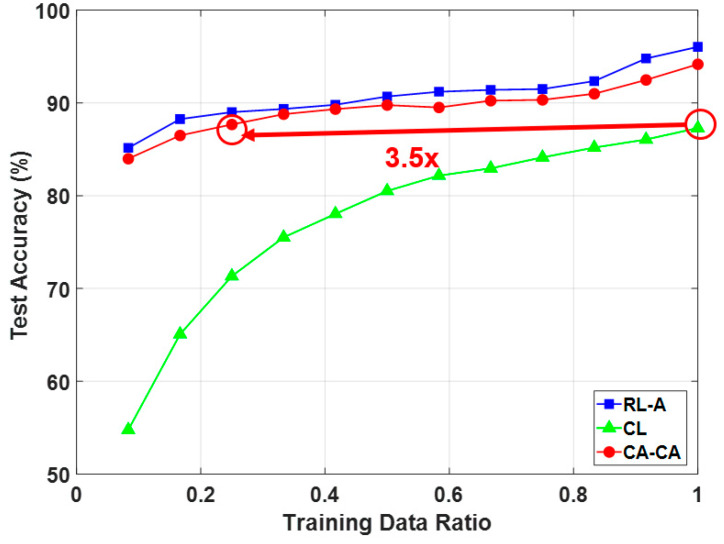
Comparison of classification accuracy under a different number of training data.

Figure 13 shows the average classification accuracy under the different numbers of training data with the compression ratio = 0.5. It can be seen that (1) our proposed compressed alignment-aided compressive analysis algorithm has a higher accuracy than compressed learning. This improvement is due to the alignment of the signal making the ML easier to learn; (2) our proposed compressed alignment-aided compressive analysis algorithm can maintain an around 90% accuracy while reducing the training data by 70%; and (3) although the accuracy of our proposed compressed alignment-aided compressive analysis algorithm is slightly lower than reconstructed learning with alignment, the proposed CA-CA framework can avoid reconstruction, and thus has a much lower computational time, which is shown in the next paragraph. 

## 5. Analysis of Computation Time and Memory Overhead 

**Figure 14:**
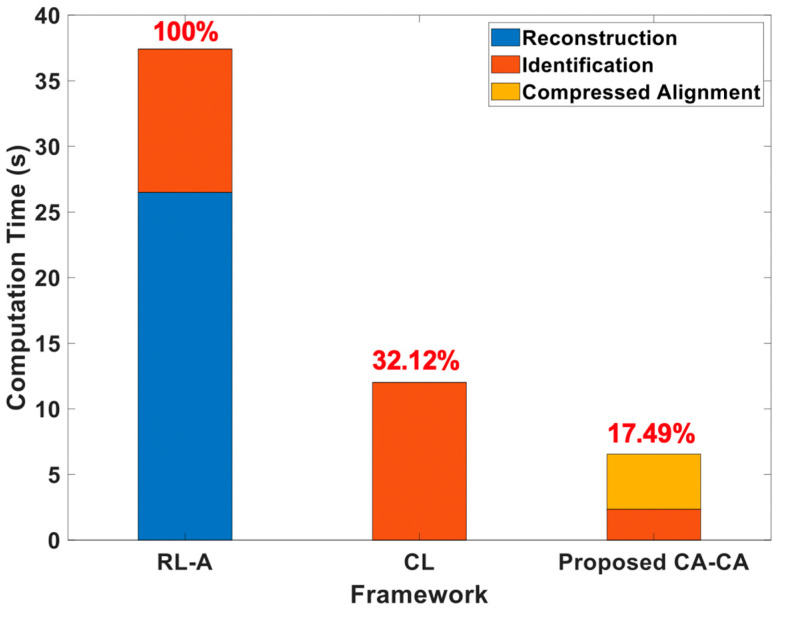
Analyses of the computational time under different frameworks.

**Figure 15:**
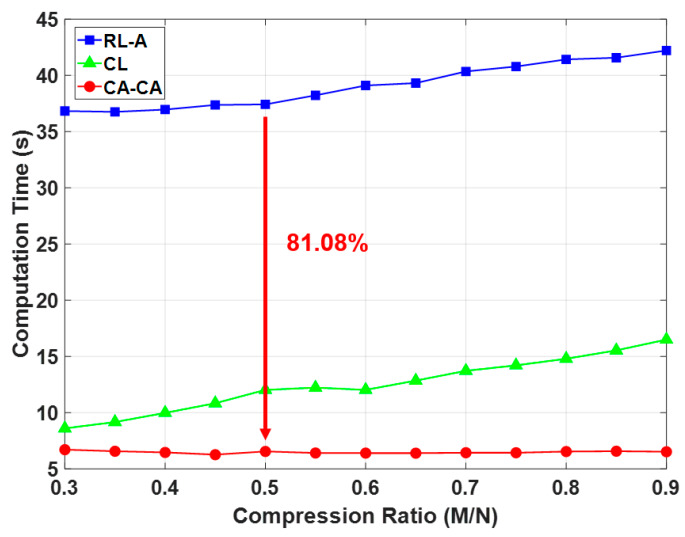
Comparison of computation time under different compression ratio.

Here, we analyzed the computation time under different frameworks, as shown in Figure 14. The simulation settings are the same as in Table 2, except that the compression ratio is 0.5 and the number of training data is 600. It can be seen that (1) the computation time of the reconstruction is much higher than others. This is also the main reason leading to the highest computation time for reconstruction learning with alignment (RL-A). Therefore, compared with RL-A, our proposed reconstruction-free framework can save more computing resources; (2) the computational time of the ML-based identification model is related to the signal dimension and signal representation of the input data. Since the ECG signals are aligned in the compressed domain, the ML-based identification model can learn more detailed information about the signals. Our proposed CA-CA not only aligns the signals in the eigenspace, but also reduces the signal dimension. Hence, the although compressed alignment-aided compressive analysis algorithm requires additional pseudo-inverse and compressed-domain alignment, it helps to reduce the computational time of the SVM model for learning the compressed-domain alignment. Therefore, even if there are fewer system blocks in compressed learning (CL), compared with CL our proposed CA-CA framework still has a lower computational time. In short, compared with RL-A and CL, the computation time of our proposed compressed alignment-aided compressive analysis algorithm is reduced by 82.51% and 45.51%, respectively. Furthermore, we verified whether the same result can be obtained under different compression ratios. Figure 15 shows the computation time under different compression ratios with the number of training data = 600. It can be seen that compared with RL-A and CL, our proposed compressed alignment-aided compressive analysis algorithm also has less computation time under different compression ratios.

After that, we analyzed the resource overheads of the memory requirements in the online stage, as shown in Table 5, where *N* is the dimension of the ECG data (input data); *M* is the dimension of the compressed data; $d_{R}$ is the dictionary length; *K* is the signal sparsity setting in the reconstruction algorithm; $k_{d}$ is the length of the PCA-based dictionary; and $n_{SV}$ is the number of support vectors in the SVM. Under the simulation settings, Table 5 also shows the accurate number of total memory requirements of the different algorithms in the online stage. 

In reconstructed learning with alignment, when the compression ratio (*M*/*N*) is 0.5, the measurement matrix ($Md_{R}$ = 125 × 250 ≅ 31.25 K) and the sparsifying matrix ($Nd_{R}$ = 250 × 250 ≅ 62.5 K) are required. A heavyweight SVM model in the time domain ($n_{SV}N$ = 4920 × 250 ≅ 1.23M) is also required. In compressed learning, when the compression ratio (M/N) is 0.5, the SVM models in the compressed domain ($n_{SV}M$ = 8689 × 125 ≅ 1.09 M) is required. Furthermore, $n_{SV}$ increases because of learnability degradation in the measurement domain where the ECG signal is unaligned. In the proposed compressed alignment-aided compressive analysis algorithm, it needs another compressed alignment resource, which is $\left(M+k_{d}\right)N\;$= (125 + 39) × 250 ≅ 41 K. In addition, it also needs to store a small projection matrix ($k_{d}M$ = 39 × 125 ≅ 4.875 K), which is computed using the above alignment resource. A lightweight SVM model in eigenspace ($n_{SV}k_{d}$ = 4095 × 39 ≅ 0.16 M) is also required. Due to the same reason as reconstructed learning with alignment, the support vector in the SVM is also reduced. Besides, compared with the reconstructed learning with alignment algorithm, $n_{SV}$ decreases due to learnability improvement in the eigenspace. Therefore, the total memory requirement of the compressed analysis (0.21 M) is 6.47 and 5.3 times fewer compared with reconstructed learning (1.33 M) and compressed learning (1.09 M), respectively.

It can be seen and proved again that (1) our proposed compressed alignment-aided compressive analysis algorithm has low complexity because there is no need to reconstruct the ECG signal; and (2) because of the good data representation (aligned ECG signals), the SVM can obtain more detailed information. Therefore, our proposed compressed alignment-aided compressive analysis algorithm has higher accuracy than compressed learning. Compared to reconstructed learning with alignment, the memory overhead of the compressed alignment-aided compressive analysis algorithm is reduced 6.5 times. Compared to compressed learning, the accuracy of the compressed alignment-aided compressive analysis algorithm is improved by 7.11%.

## 6. Conclusions

In this work, we introduced the compressed-domain ECG biometric user identification. The resources in wearable devices are very limited. However, CS reconstruction makes the complexity of the biometric system higher. On the other hand, the aligned ECG signal can perform better user identification performance. However, it is difficult to align the compressed signal because its characteristics are corrupted. Therefore, we propose a compressed-domain alignment-aided compressive analysis to directly align the ECG signal from the compressed signals without reconstruction. In this algorithm, we reduced the training data ratio by 70% due to the alignment. The required computation time is reduced by 81.08% due to the absence of reconstruction. This work shows the first step to demonstrate the feasibility and effectiveness of using compressed data for ECG-based user identification. At present, we have only a small ECG data base (with ID information) to verify our algorithm. In future work, prospective researchers can augment our algorithm in various situations by applying it to different physiological signals and by using larger databases.

## Figures and Tables

**Table 1:** Qualitative comparison of the complexity and performance of different frameworks.

Framework	Complexity (Time)	Performance (Accuracy)
Reconstructed Learning with Alignment	High	High
Compressed Learning	Low	Low
Proposed Algorithm	Very Low	High

**Table 2:** Simulation settings.

Simulation Program	MATLAB
Processor Configuration	
Core	Intel i7-7700 with 8 GB RAM
Data Parameters	
Source	PhysioNet QT-Database
Sampling Frequency	250 Hz
Input Dimension (N)	250 (1 s ECG)
Number of Classes	22 users
Number of Training/Inference Data	(50 to 600)/250 each class
Size of the Dictionary Ψ	Mx39
CS Sensing Matrix Φ	
Type	Random Gaussian
Compression Ratio (M/N)	(0.9 to 0.1)
Machine Learning Model	
Type	RBF Kernel SVM by LIBSVM [29]
Cost (C) Search Range	1, 5, 10
Gamma (γ) Search Range	($1\times 10^{-4}$ to 1)
Cross-Validation	5-fold

**Table 3:** Dictionary size and accuracy of the different algorithms *.

Framework	Dictionary Size	Testing Accuracy (%)
Reconstructed Learning	250 × 250 (100%)	87.89
Compressed Learning	No Dictionary	87.05
Proposed Compressive Analysis	250 × 39 (15.6%)	87.67

* Compression ratio = 0.5.

**Table 4:** Computation time and accuracy of the different algorithm *.

Framework	Accuracy	Computation Time
Reconstructed Learning with Alignment	96.03%	37.41 s
Compressed Learning	87.05%	12.02 s
Proposed Compressed Alignment-aided Compressive Analysis	94.16%	6.55 s

* Compression ratio = 0.5, Number of training data = 600 (each class).

**Table 5:** Memory overheads of the different frameworks.

Framework	Memory Requirement (Formula)	Memory Requirement (Accurate Number)
Reconstructed Learning with Alignment	$\left(M+N\right)d_{R}+n_{SV}N$	1.33 M
Compressed Learning	$n_{SV}M$	1.09 M
Proposed Algorithm	$\left(M+k_{d}\right)N+k_{d}M$ $+n_{SV}k_{d}$	0.21 M
